# Psychosocial burden of type 1 and 2 hereditary angioedema: a single-center Canadian cohort study

**DOI:** 10.1186/s13223-021-00563-0

**Published:** 2021-06-29

**Authors:** Julia Hews-Girard, Marilyn Dawn Goodyear

**Affiliations:** 1 Southern Alberta Rare Blood and Bleeding Disorders Comprehensive Care Program, Calgary, AB T2N 2T9 Canada; 2 Cumming School of Medicine, University of Calgary, Calgary, AB T2N 1N9 Canada

**Keywords:** Hereditary angioedema, Depression, Anxiety, Stress, Health-related quality of life, Work productivity, Activity impairment, Disease burden

## Abstract

**Background:**

Hereditary angioedema (HAE) is a rare but serious disorder associated with a multifaceted burden of illness including a high prevalence of psychiatric symptoms and impaired health-related quality of life (HRQoL). Despite recent efforts to clarify the psychosocial implications of HAE, important gaps still remain. The aim of this study was to characterize the psychosocial burden associated with HAE types 1 and 2.

**Methods:**

Type 1 or 2 HAE patients (n  =  17), aged 19 years or older, completed the Depression, Anxiety, Stress Scale (DASS-21) and the DSM-5 cross cutting measures to identify psychiatric symptomatology, Angioedema Quality of Life Questionnaire (AE-QoL) and the Short-Form 36-Item Health Survey version 2 (SF-36v2) to assess disease-related and generic HRQoL respectively, and the Work Productivity and Activity Impairment Questionnaire (WPAI) to measure impact on work productivity and daily activities. Data analyses were conducted using SPSS statistical software (Version 25.0; IBM, Armonk, NY). Descriptive statistics were used to summarize continuous demographics and clinical characteristics and outcomes of interest while frequency distributions were used for categorical variables. T tests were used to compare SF-36v2 domain scores to Canadian norms and sex differences in scale scores.

**Results:**

Depression [DASS-21 score  =  6.8  ±  10.2; n  =  12 (71%)] anxiety [DASS-21 score  =  6.2  ±  8.2; n  =  13 (76%)] and stress [DASS-21 score  =  10  ±  10.2; n  =  13 (76%)] were prevalent. Other psychiatric symptoms warranting inquiry included mania (n  =  14, 82.4%), anger (n  =  14, 82.4%), sleep disturbances (n  =  13, 76.5%), somatic symptoms (n  =  11, 64.7%) and impaired personality functioning (n  =  9, 52.9%). Mean AE-QoL score was 39  ±  18.2. Mean SF-36v2 domain scores were significantly lower than Canadian normative data for the entire sample (p  <  0.05). Impairment in work productivity was minimal; mean activity impairment was 20.6%  ±  21.1% [n  =  11 (64.7%)]. Female participants reported significantly greater HAE-related stress [DASS; t(15)  =   − 2.2, p  =  0.04], greater HAE-related fears [AEQoL; t(5.6)  =   − 2.7, p  =  0.04), and lower SF-36v2 domain scores than male patients.

**Conclusions:**

Study findings offer specific, valuable insight into the psychosocial burden of HAE with the potential to improve clinical management of HAE. Best practices for effective management of HAE should include providing holistic care to address the psychosocial and mental health of HAE patients.

## Background

Types 1 and 2 hereditary angioedema (HAE) are rare genetic disorders arising from deficiency (type 1) or dysfunction (type 2) of the C1-inhibitor (C1-INH) protein [1]. HAE is characterized by edematous swelling attacks of the face, extremities, abdomen and genitalia and potentially life-threatening laryngeal swelling mediated by excessive production of bradykinin [2, 3]. HAE attacks are painful, occur with unpredictable frequency [4] and can require emergency medical attention. In addition, diagnosis is often delayed, with patients undergoing unnecessary medical procedures and ineffective treatment. These factors can lead to a continuous fear of attacks and psychological distress [5, 6]. Limited evidence suggests that depression and anxiety are prevalent in patients with HAE; however, the mental health sequelae of HAE remain largely underrecognized and untreated.

The disease burden associated with HAE is multifaceted. HAE symptoms can be debilitating and can also negatively affect patients’ ability to perform daily activities and productivity at work or school [5, 7, 8]. Furthermore, individuals with HAE consistently report poor health-related quality of life (HRQoL) across multiple dimensions. While improvements have been shown with recent advances in treatment, characterization of the psychosocial burden of illness associated with HAE is an unmet medical need for the optimal management of patients with HAE.

The present study aimed to characterize the psychosocial burden in a Canadian cohort with HAE types 1 and 2 and to explore the impact of HAE on quality of life and work productivity, using validated generic and HAE-specific tools.

## Methods

### Study participants

The present study was a single-center, cohort study of patients  ≥  18 years old with HAE type 1 or 2, followed at the Southern Alberta Rare Blood and Bleeding Disorders Comprehensive Care Program. As the diagnosis of HAE with normal C1 level and function (nC1-INH) cannot be confirmed with standardized testing, individuals with this diagnosis were excluded. Eligible participants completed questionnaires assessing generic and HAE-related mental health, quality of life and work productivity. The study was approved by the University of Calgary Ethics Board (REB# 17-0542), and all participants provided written informed consent prior to any study procedures.

### Assessments

Demographics and medical history, including HAE diagnosis and treatment regimen, were collected. Psychosocial outcomes and HRQoL were assessed using validated patient-reported questionnaires. Symptoms of depression, anxiety and stress were assessed using the Depression, Anxiety and Stress Scale (DASS-21) [9]. The DASS-21 consists of three 7-item questionnaires that assess depression, anxiety, and stress experienced over the last 7 days, using a 4-point severity scale.

Additional mental health domains that may have a significant impact on patient treatment and prognosis were identified using The Diagnostic and Statistical Manual of Mental Disorders Ed. 5 Level 1 Cross-Cutting Symptom Measure [10]. This measure of current mental health symptomatology is composed of 23 items assessing 13 psychiatric symptoms, including depression, anger, mania, anxiety, somatic symptoms, suicidal ideation, psychosis, sleep problems, memory, repetitive thoughts and behaviors, dissociation, personality functioning, and substance use during the prior 2 weeks. The bothersomeness of each item is rated on a 5-point scale; need for additional inquiry is determined by the highest score on any item within a domain.

Disease-specific HRQoL was assessed using the Angioedema Quality of Life Questionnaire (AE-QoL) [11]. The AE-QoL is a 17-item questionnaire that assesses four dimensions (functioning, fatigue/mood, fears/shame, and nutrition) to determine the impact of angioedema on daily life over the course of the prior 4 weeks. Scores were transformed to a linear scale of 0–100; a scale was used to define the severity of QoL impairment (0  =  none; 0–25  =  mild; 26–75  =  moderate and  >  75  =  severe).

The Medical Outcome Study Short-Form 36-Item Health Survey version 2 (SF-36v2) [12] was used to measure generic HRQoL in eight health domains including physical functioning (PF), limitations in daily role functioning due to physical problems (RP), bodily pain (BP), general health (GH), vitality (VT), social functioning (SF), limitations in daily role functioning due to emotional problems (RE) and mental health (MH) based on the patients’ experiences over the last 4 weeks. Scores for each domain range from 0 to 100 with higher scores indicating better HRQoL.

Work productivity was determined using the Work Productivity and Activity Impairment Questionnaire (WPAI) [13]. This 6-item questionnaire assesses the impact of health problems on the patients’ ability to work and participate in activities of daily living based on their experiences over the prior 7 days. The four domains measured include absenteeism (work time missed due to health problems), presenteeism (work impairment due to health problems), work productivity loss (overall work impairment due to health problems), and activity impairment (impairment in regular activities due to health problems).

### Outcomes

The present study primarily sought to determine the magnitude of the negative emotional states of depression, anxiety, and stress as measured by DASS-21 scores in patients with HAE. The proportion of HAE patients with mild, moderate, and severe symptoms were also reported. In addition, disease-related and generic HRQoL were examined using the AE-QoL and SF-36v2 tools, respectively. Work productivity was assessed by the WPAI, and mental health domains were assessed using the DSM-5 Level 1 Cross-Cutting Symptom Measure.

### Statistical analysis

Data analyses were conducted using SPSS statistical software (Version 25.0; IBM, Armonk, NY). Descriptive statistics were used to summarize continuous demographics and clinical characteristics and outcomes of interest while frequency distributions were used for categorical variables. T tests were used to compare SF-36v2 domain scores to Canadian norms and sex differences in scale scores.

## Results

### Patient characteristics

Demographics and clinical characteristics of all patients (n  =  17) are reported in Table 1. The majority were female (n  =  13; 76%) with a diagnosis of type 1 HAE (n  =  11; 64.7%). Regarding individual treatment protocol, 11 (64.7%) participants reported using prophylaxis, compared to 6 (35.3%) reporting on-demand treatment. Six participants [4 using prophylaxis] reported having 6–10 attacks in the 12 months prior to study, while 11 participants (7 using prophylaxis) reported  <  6 attacks. All except one participant (94.1%) self-administered treatment.

**Table 1:** Demographics and clinical characteristics (n  =  17). Values are represented in mean [range] or n (%)

	Total population	Patients using prophylaxis	Patients using on-demand treatment
	17	11	6
Age	43 [20–63]	–	–
Sex			
Male	4 (24%)	3	1
Female	13 (76%)	8	5
Diagnosis			
Type 1	11 (64.7%)	8	3
Type 2	6 (35.3%)	3	3
Treatment administration			
Self-administered treatment	16 (94.1%)	11	5
Provider administered treatment	1 (5.9%)	0	1
Number of attacks in 12 months prior to study			
6–10	6	4	2
< 6	11	7	4
Work status			
Unemployed	4	2	2
Retired	1	0	1
School (full-time)	1	0	1
Employed part-time	5	3	2
Employed full-time	6	5	1

### Negative emotional states

#### Depression

**Fig. 1:**
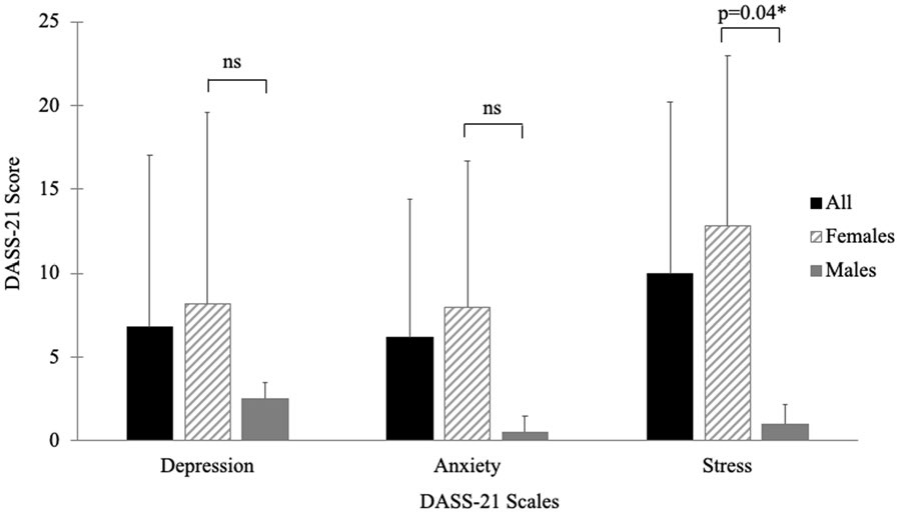
Depression, anxiety and stress in patients with HAE. Mean DASS-21 depression score in all participants was 6.8  ±  10.2. Mean DASS-21 anxiety score in all participants was 6.2  ±  8.2. Mean DASS-21 stress score in all participants was 10  ±  10.2. Female participants (hashed line bar) reported significantly greater HAE-related stress compared to male participants (gray bar) [t(15)  =   − 2.2, p  =  0.04]

As shown in Fig. 1, mean DASS-21 depression score in all participants was 6.8  ±  10.2. Of 12 (71%) participants reporting depression, none reported moderate symptoms, but 3 (18%) reported severe depressive symptoms.

#### Anxiety

Mean DASS-21 anxiety score in all participants was 6.2  ±  8.2 (Fig. 1). Of 8 (47%) participants reporting anxiety, 4 (24%) reported mild symptoms, and 4 (24%) reported moderate to severe symptoms.

#### Stress

Mean DASS-21 stress score in all participants was 10  ±  10.2 (Fig. 1). Of 13 (76%) participants reporting stress, 4 (24%) reported mild stress, 5 (29%) reported moderate stress, and 4 (24%) reported severe stress. Female participants reported significantly greater HAE-related stress compared to male participants [t(15)  =   − 2.2, p  =  0.04].

There was no significant difference in depression, anxiety, or stress scores between participants on prophylaxis versus on-demand treatment. There was also no significant difference in these scores when comparing participants by attack frequency (i.e., 6–10 attacks in the past 12 months versus  <  6 attacks in the past 12 months).

### Comorbid psychiatric symptomatology

**Fig. 2:**
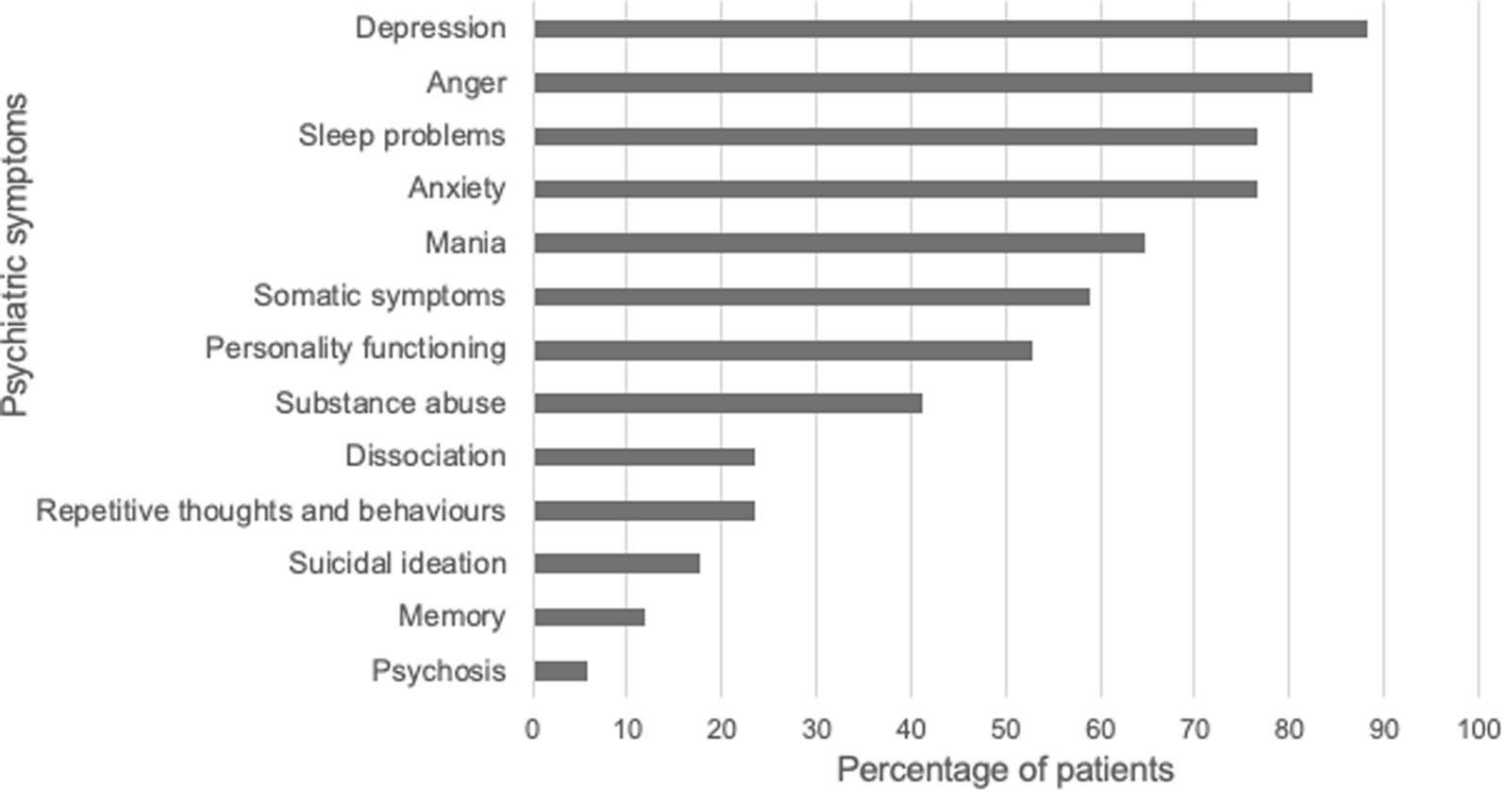
DSM-5 Level 1 cross-cutting symptom measure outcomes reported by patients with HAE. Of the 13 psychiatric symptoms evaluated, more than half the participants reported experiencing the following psychiatric symptoms warranting further exploration: depression (n  =  15, 88.2%), anger concerning the disease (n  =  14, 82.4%), sleep disturbances (n  =  13, 76.5%), anxiety (n  =  13, 76.5%), mania (n  =  11, 64.7%), somatic symptoms (n  =  10, 58.8%) and impaired personality functioning (n  =  9, 52.9%)

As shown in Fig. 2, the most commonly reported psychiatric symptoms warranting further exploration were depression (n  =  15, 88.2%), anger concerning the disease (n  =  14, 82.4%), sleep disturbances (n  =  13, 76.5%), anxiety (n  =  13, 76.5%), mania (n  =  11, 64.7%), and somatic symptoms (n  =  10, 58.8%). Participants also frequently reported impaired personality functioning (n  =  9, 52.9%), which encompasses not knowing who they are or what they want out of life, or not feeling close to others or enjoying relationships.

### Health-related quality of life

#### Disease-related QoL

**Fig. 3:**
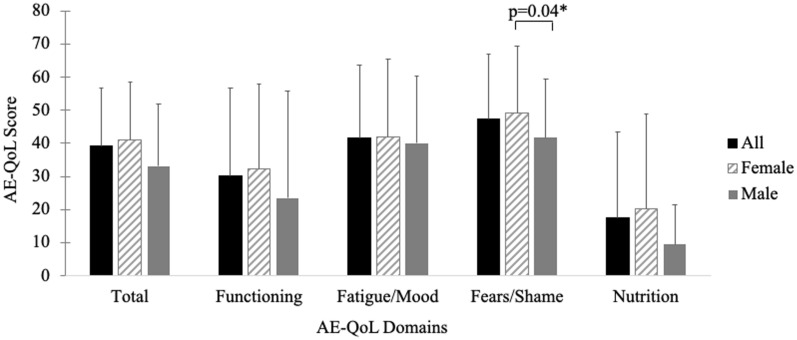
AE-QoL domain scores in patients with HAE. Mean total AE-QoL score in all participants was 39 ± 18.2 indicative of moderate impairment in HRQoL due to HAE. Moderate impairment in the health-related fatigue (mean score  =  40.3  ±  22.7) and fear (mean score  =  47.2  ±  20.7) domains was also reported. Female participants (hashed line bar) reported significantly greater HAE-related fear, compared to male participants [gray bar; t(5.6)  =   − 2.7, p  =  0.04]

All participants experienced poor overall HRQoL. As shown in Fig. 3, mean  ±  SD total AE-QoL score of 39  ±  18.2 indicates moderate impairment in HRQoL due to HAE. Mean  ±  SD scores of 40.3  ±  22.7 and 47.2  ±  20.7 in health-related fatigue and fear, respectively, are also consistent with moderate impairment. Female participants reported significantly greater HAE-related fear, compared to male participants [t(5.6)  =   − 2.7, p  =  0.04].

#### Generic HRQoL

**Fig. 4:**
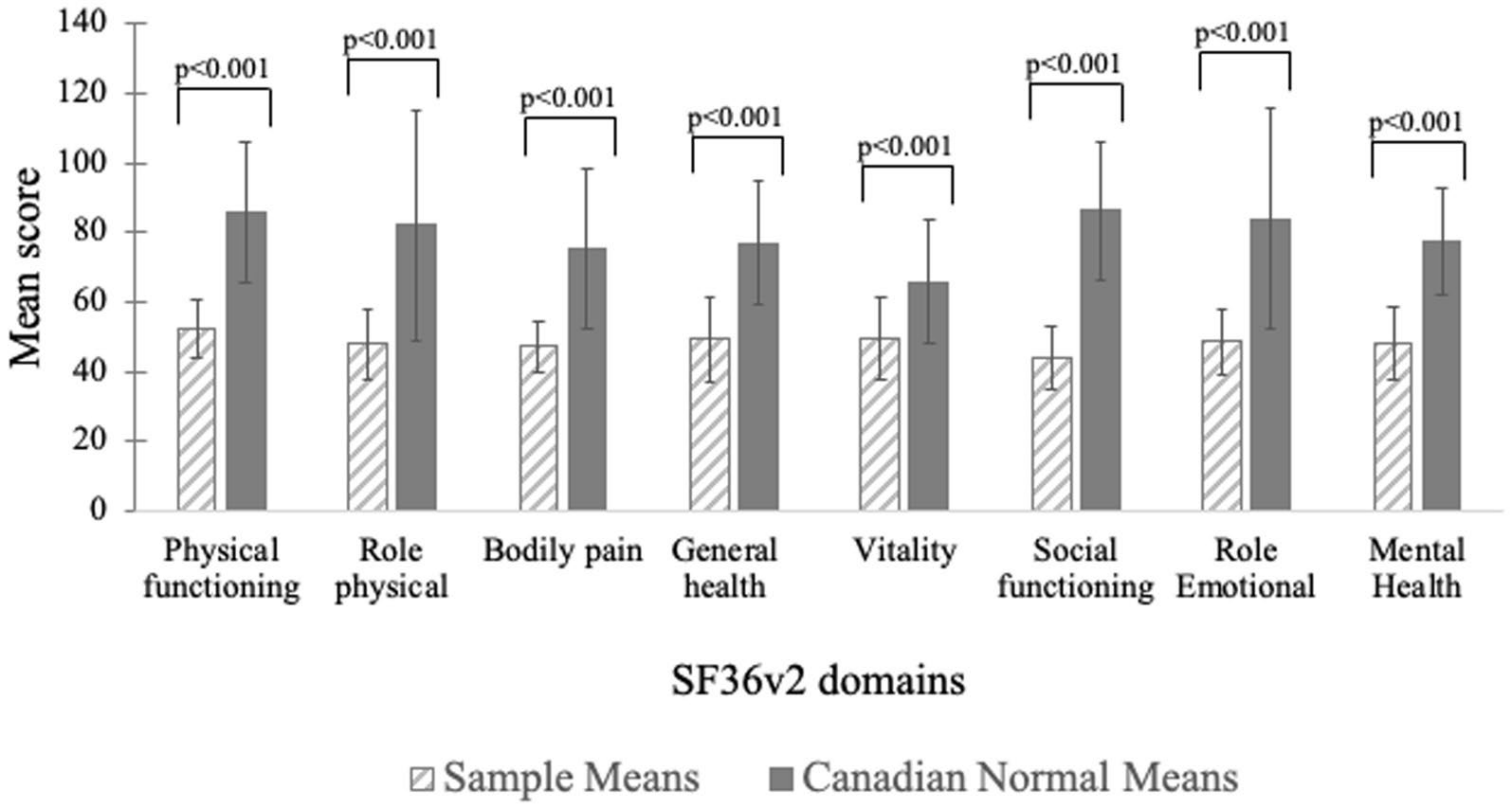
HAE cohort SF-36v2 scores compared to Canadian normative scores. Mean scores of all domains of the SF-36v2 in the study sample (hashed line bar) were significantly lower than Canadian normative data (gray bar; p  <  0.001 for all). The greatest deficits were seen in physical functioning, social functioning, role physical and role emotional domains

As shown in Fig. 4, mean scores of all domains of the SF-36v2 were significantly lower than Canadian normative data for the entire sample (p  <  0.001 for all). Specifically, PF and SF, as well as RP and RE scores of the SF-36v2, were the most impacted. Female participants reported significantly lower scores than male patients in most SF-36v2 domains (data not shown).

### Work productivity

Most survey participants (64.7%) were employed (Table 1). Only one participant reported work absenteeism of 31.1% due to health problems, which translated into a work productivity loss of 27%. Mean presenteeism reported was 10%  ±  18% (n  =  5). A total of 11/17 (64.7%) participants reported impairment in activities of daily living due to health problems; mean activity impairment reported was 20.6%  ±  21.1%.

## Discussion

The present study characterizes the psychosocial burden of HAE in a single cohort. Most participants experienced depression, anxiety, and stress. Preliminary results from this study also indicate high rates of coexistent psychiatric symptomatology warranting further evaluation. Furthermore, participants reported poor HRQoL and impairment in regular activities associated with HAE.

Participants were primarily female, which aligns with published literature supporting a clear female predominance for types 1 and 2 HAE [14]. Most participants (7/11) with a lower frequency of attacks (<  6 attacks in the past 12 months) were using prophylaxis treatment. However, there was no significant difference found between treatment type and psychosocial scores. As prophylaxis use is more common in more severe HAE cases, it is difficult to disentangle the participant’s current medical condition from past experience with respect to psychosocial burden. The apparent lack of correlation between prophylaxis use and psychosocial scores also highlights the need for psychosocial intervention, as medical treatment alone may not improve an individual’s psychosocial health.

The prevalence of depression in the present sample was higher than rates reported in previous cohort studies (14–42.5%) [5, 15–17], whereas prevalence of anxiety (~  50%) was comparable to previous findings [16, 17]. Differences in rates of depression between our sample and others may be due to different methodology used to determine the prevalence of depression in study samples; in some studies, patients completed self-report scales [5, 16, 17], whereas structured scales were administered by trained study personnel in other studies [15].

The study provides suggestive evidence of high rates of co-existent psychiatric symptomatology that remains underrecognized in patients with HAE, including mania, anger, sleep disturbances, somatic symptoms, and impaired personality functioning. Notably, sleep disturbances do not appear to have been reported previously in patients with HAE. We postulate that the high prevalence of sleep disturbances reported in this study may be associated with HAE attacks starting or evolving during the night, or may be a consequence of psychological distress, as previously reported [15]. The high measures of mania symptomology also overlapped with the reported sleep disturbances, as the mania domain involves reports of less sleep but high energy levels, in addition to increased risk-taking/starting more projects. Overall, these findings suggest psychiatric symptomology of interest for additional research in the context of HAE burden. More immediately, they suggest that physicians treating patients with HAE should routinely inquire about mental health and refer patients to specialists when needed.

The poor generic HRQoL scores in this study were consistent with previous reports of significantly diminished HRQoL compared with population norms [16, 17], even between attacks [5]. Specifically, previous studies reported that physical health and social functioning were the most affected, which is similar to findings from the present study [16, 18]. Poorer scores in the MH domain imply that negative emotional states are associated with poorer quality of life in patients with HAE, while poorer HRQoL scores across the PF and BP domains suggest that patients may experience somatic symptoms that may be secondary to depression and/or other negative emotional states. Furthermore, all patients reported poor generic HRQoL even though they were asymptomatic at the time of the survey. There was no correlation between reduced attack frequency and improved psychosocial well-being, which suggests that impaired HRQoL may be slow to resolve, despite clinical improvement.

The impact of HAE on school or work was minimal in the present cohort, in contrast to previous studies that have generally reported absenteeism, decreased work productivity, and activity impairment in patients with HAE [5, 17, 18]. For instance, in a large US-based survey study, 57.5% of patients reported that HAE symptoms negatively affected career advancement [17]. Likewise, a European survey study showed that 36% of patients reported that HAE symptoms prevented career advancement and 42% of patients reported hindered educational advancement due to HAE [18]. In the present study, a wide variability in productivity loss was observed, ranging from no loss to up to 70% loss. A registry study in Sweden with similar variability in absenteeism related to HAE symptoms found that the degree of impact may be associated with severity of attacks [19]. Previous findings also suggest that treatment regimen may be associated with outcomes such as work productivity; studies with a greater proportion of patients using prophylaxis for the prevention of HAE attacks [17], similar to the present study, report less absenteeism, presenteeism, work productivity loss and activity impairment compared to those with a greater proportion of patients using on-demand treatment [18]. These findings imply that prophylactic treatment may be of particular benefit in HAE patients experiencing work productivity and activity impairments.

It has been previously suggested that negative emotional states in patients with HAE may partly be a secondary response to chronic disease burden associated with delays in diagnosis and unnecessary surgical procedures in patients with HAE [7]; however, emerging evidence in-vivo suggests that pathophysiological mechanisms responsible for HAE may also partly account for the increased risk of negative emotional states and somatic symptoms in this patient population. Specifically, deficiency or lack of C1-INH protein in patient with HAE leads to the excessive production of the vasoactive peptide bradykinin, which mediates the symptoms characteristic of HAE [20, 21]. Bradykinin circulates at higher-than-normal levels in HAE patients, even between attacks, and the breakdown product of bradykinin, des-arg-9-bradykinin, is a strong agonist for bradykinin B1 receptors, which have been reported to directly mediate depressive symptoms in animal models [22–24]. Increased vessel permeability mediated by bradykinin may be amplified by the cytokine IL-1β, which has been documented to regulate behaviour [25, 26]. While inflammation is not directly related to the characteristic swelling seen in HAE, animal studies suggest that inflammatory pathways involving IL-1 β may be stimulated in the absence of C1-INH, possibly leading to depressive symptoms [25, 26] and exacerbation of HAE attacks [15, 27]. Therefore, negative emotional states and physical symptoms between attacks may be secondary to chronic disease burden, functional C1-INH deficiency, or both. Studies showing improvements in both physical and emotional functioning and quality of life with use of C1-INH for the prevention of HAE attacks compared to placebo suggest that prophylactic treatment may be a promising strategy to manage various HRQoL deficits associated with HAE [28, 29]. Future research should elucidate the poorly characterized effects of HAE on patients’ emotional and physical states and explore interventions that can ameliorate the burdens associated with this disease.

## Conclusions

This Canadian study found a high prevalence of psychiatric symptomatology, poor HRQoL and impairments in activities of daily living in patients with HAE. Women with HAE appeared to be particularly affected. The present study adds to the growing body of literature aiming to better understand the burden of illness associated with HAE; however, gaps still remain in disease management. Study findings suggest that physicians treating patients with HAE should routinely inquire about mental health and psychosocial well-being and refer patients to specialist care as appropriate. Providing holistic care to patients with HAE, including managing symptoms reported by patients such as depressive and anxiety symptoms, stress and sleep disturbances, may be important for improving outcomes. Future evaluation should include larger samples and further investigation of gender differences.

## Data Availability

The datasets generated and/or analyzed during the current study are not publicly available since participants did not provide consent for their data to be shared publicly.

## Abbreviations

AE-QoL: Angioedema Quality of Life Questionnaire
BP: Bodily pain domain of the SF-36v2
C1-INH: C1-inhibitor
DASS-21: Depression, Anxiety and Stress Scale
GH: General health domain of the SF-36v2
HAE: Hereditary angioedema
HRQoL: Health-related quality of life
MH: Mental health domain of the SF-36v2
PF: Physical functioning domain of the SF-36v2
RE: Limitations in daily role functioning due to emotional problems domain of the SF-36v2
RP: Limitations in daily role functioning due to physical problems domain of the SF-36v2
SF: Social functioning domain of the SF-36v2
SF-36v2: Medical Outcome Study Short-Form 36-Item Health Survey version 2
VT: Vitality domain of the SF-36v2
WPAI: Work Productivity and Activity Impairment Questionnaire
